# Changes in Crisis Resolution Home Treatment Team Referral Numbers and Patient Caseload During COVID-19 Pandemic in First Lockdown

**DOI:** 10.1192/bjo.2022.477

**Published:** 2022-06-20

**Authors:** Awon Raza, Sujith Pillai

**Affiliations:** Cambridgeshire and Peterborough NHS Foundation Trust, Peterborough, United Kingdom

## Abstract

**Aims:**

The COVID-19 Pandemic has had an impact on most aspects of functioning on the world in general. We wanted to see what impact of COVID-19 Pandemic has had on a Crisis Resolution Home Treatment Team North Peterborough. The main objectives of this audit were to see changes in Crisis Resolution Home Treatment Team North number and source of referrals, average length of stay, total number of patients Home Treated during this period (Pre and during COVID-19 pandemic) and to also identify whether patients with a certain diagnosis deteriorated or presented more to services compared to others.

**Methods:**

We retrospectively reviewed case-notes and data were collected from RiO Electronic Patient Records (EPR) covering all the factors we wanted to analyse. Data collection periods were pre-COVID-19 between 1st April 2019 and 30th September 2019 and COVID-19 pandemic (1st Lockdown) between 1st April 2020 and 30th September 2020. Total number of referrals received between April and September 2019 pre-pandemic were 844 and total number of referrals received during COVID-19 pandemic between April and September 2020 were 660. Data were exported from the electronic patient record into Microsoft Excel and quantitative analysis was performed using Microsoft Excel.

**Results:**

The results showed 21.8% drop in total number referrals from 844 to 660 and there were 20.89% (79) less patients Home Treated from April and September 2020 during first lockdown compared to the similar period in 2019. Significant increase observed in patients with bipolar affect illness by 32% (from 86 to 128 patients), acute stress reaction and adjustment disorder by 15% (from 68 to 80 patients) and psychotic disorder by 11.5% (from 245 to 277patients) in 1st lockdown period compared to 2019 similar period. Declining trend observed in intentional self-harm by various means by 20.75% and 4% drop in personality disorder patients. Anxiety and depression patients number remained same in both periods.

**Conclusion:**

Although referral numbers dropped significantly and Crisis Resolution and Home Treatment Team caseload decreased during the COVID-19 pandemic first lockdown, the number of patients with serious mental illness presented to services increased remarkably (bipolar and psychotic illness). Overall, no major change in length of stay of patients with Crisis Team was observed when compared both periods and referral numbers remained low from all sources during COVID-19 pandemic.

